# Dietary Calcium Intake in Sample of School Age Children in City of Rabat, Morocco

**DOI:** 10.1155/2018/8084623

**Published:** 2018-04-08

**Authors:** Amina Bouziani, Naima Saeid, Hasnae Benkirane, Latifa Qandoussi, Youness Taboz, Asmaa El Hamdouchi, Khalid El Kari, Mohammed El Mzibri, Hassan Aguenaou

**Affiliations:** Joint Unit of Nutrition and Food Research, CNESTEN–Ibn Tofaïl University–URAC 39, Regional Designated Center for Nutrition (AFRA/IAEA), Rabat, Morocco

## Abstract

Calcium is an important mineral playing a vital role to maintain bone health. Calcium intake is considered as one of the most important determinants to assess the calcium status and to evaluate the calcium deficiency in the human body. Our study aims at estimating calcium intake in a sample of children and adolescent to be used in the global strategy to reduce calcium deficiency disorders in Morocco. Thus, 131 children and adolescents were recruited from public schools at Rabat and its regions in the framework of a descriptive cross-sectional study. For each participant, anthropometric parameters were measured. Calcium status was assessed by 24 h dietary recall. Food frequency questionnaire was used to evaluate children's nutritional habits and to assess the consumption of calcium-rich food. Overall, the mean calcium intake was 522.0 ± 297.0 mg/day, and 85.5% of subjects highlighted calcium deficiency, with no significant difference between boys and girls. Calcium intake was significantly different according to age groups, and high consumption of calcium was found in subjects aged from 14 to 18 years (776.86 ±290.07 mg/day), giving evidence of the low calcium status of the studied population. Daily food intake and food frequency analysis showed that bread, vegetables, and fruits are the most consumed food and the main source of daily calcium intake. Consumption of dairy products, considered as the best source of calcium, is lower and represents only 14% of total calcium intake. Our study clearly showed that calcium status is very lower in Moroccan children and adolescents and a large proportion of this population have inadequate calcium intake. Hence, there's an urgent need of specific strategies, including children sensitisation and nutritional education, to increase calcium intake and therefore reduce calcium deficiency disorders impacting the whole body during childhood and in adult age.

## 1. Introduction

Calcium is the most abundant mineral element in the body, representing 1–2% of total body weight [1]. Overall, 99% of calcium is found in the skeleton and teeth, as calcium phosphate, providing rigidity by virtue of the insoluble salts it forms with phosphoric acid, the remaining 1% of calcium is freely exchangeable with the extracellular fluid [2]. The concentration of calcium in the organism is systematically regulated by parathyroid hormones, vitamin D, and calcitonin [3], and calcium deposition is depending on many factors, especially age, sodium status, and the presence of some animal proteins [4].

Indeed, calcium deposition on bones is dependent on the growth rate: from birth until the age of 30 years, about 150 mg of calcium per day is accumulated in the skeleton [5], during maturity (30–50 years) calcium absorption is variable and depends on calcium intake, and at elderly age (more than 50 years), the calcium balance becomes negative and bones are more likely to loss calcium [6]. Therefore, there's evidence that calcium intake during childhood and adolescence is critical, and adequate calcium intake during this period of life cycle is of a great interest to have a positive calcium balance, good bone density necessary for the skeletal consolidation [7], and reducing the risk of fractures and osteoporosis later [8].

In childhood, one of the most important nutritional risks is poor eating habits, including skipping meals, eating lots of processed food, and following fad diets [9], factors that promote an inadequate calcium intake and consequently growth problems in children and adolescents [10]. Calcium is present in many foods, but milk and dairy products, such as yogurt, cheeses, and buttermilk, are the best source of calcium (∼1150 mg/l), which is more available and easily absorbed in the organism. Calcium is also present in some vegetables like flour, beans, eggs, and fish. Mineral drinking water represents an alternative source of calcium for some groups of the population [11].

Worldwide recommendations for calcium intake vary depending on age, gender, and according to some genetic and environmental factors. Accordingly, IOM, FAO, and FNB recommendations outline the importance of high calcium intake to improve bone mineral density. The adequate intake of calcium is about 1000 mg/day for children aged from 6 to 8 and about 1300 mg/day for those aged from 9 to 18 and for adolescents [12].

The Moroccan diet, basically Mediterranean, is based on a large consumption of cereals, fruits, and vegetables [13]. However, the consumption of dairy products is still limited [14]. In Morocco, the prevalence of osteoporosis is quite higher. In 2013, more than 35% of women over 50 years and about 60% of women over 60 years have been reported developing osteoporosis [15]. Moreover, more than 4300 hip fractures were recorded in 2010 giving an incidence of 60.6 per 100,000 women (95% CI: 55.1–65.6) [16].

In Morocco, the Ministry of Health has adopted a global strategy to prevent health problems related to micronutrient deficiencies [17]. However, limited information is available regarding calcium intake. Owing to the importance of calcium for both children and adults, the evaluation of the dietary intake of this mineral, especially during childhood, is very important. Therefore, we have planned to evaluate the calcium intake in Moroccan children and adolescents aged from 6 to 18 years, living in Rabat and regions, to adapt the national strategy and to prevent all calcium deficiency associated diseases.

## 2. Materials and Methods

### 2.1. Subjects and Study

This study is a descriptive cross-sectional survey. It was carried out on healthy children and adolescents aged from 6 to 18 years, recruited from primary and secondary schools located in Rabat and its regions. The study protocol was approved by the Ethical Committee Biomedical research of the Faculty of Medicine and Pharmacy, Rabat, and written informed consents were obtained from each parent of recruited child.

A total of 131 children and adolescents were recruited for this investigation. For each participating children and adolescent, information about age, sex, medical history, medication, and use of supplements of calcium or vitamin D were collected using a specific questionnaire. Interviews with all subjects' parents were performed to complete information given by children and adolescents. Thereafter, all participants were subject to anthropometric measurements and completed 24 h food recall and food frequency questionnaires.

### 2.2. Anthropometry

Anthropometric measurements were performed according to standard procedures [18]. Measurements were taken in the morning during a clinic visit by trained operators. The body weight of participating subjects, dressed in light suits, was measured with a mechanical scale (150 ± 0.1 kg, Seca 750, Germany). Standing height was taken barefoot, using a stadiometer (200 ± 0.1 cm, Seca 217, Germany). BMI was calculated as weight in kilogram divided by the square of height in meter (Kg/m2).

BMI-for-age (*z*-score), weight-for-age (*z*-score), and height-for-age (*z*-score) were then calculated using the WHO Anthro software [19]. Accordingly, BMI-for-age z-score was used to classify children into four categories: overweight: >+1SD; obesity: >+2SD; thinness: <−2SD; and severe thinness: <−3SD.

### 2.3. Dietary Intake Assessment

Dietary survey was conducted using 24 h recall protocollargely used around the world to evaluate dietary intake [20]. In this study, the multiple-pass approach was used to validate the 24 h recall questionnaire. The principle of this method consists of 3 steps:

- In the first one, participants report all food and drinks consumed in the previous day.
- In the second pass, the interviewer aims to collect more details on how the food and drinks are consumed (exact time, quantity …).
- In the third pass, the interviewer encourages the participant to memorize well by reviewing the various foods cited to remove any ambiguity [21].

For children under 12 years, parents and their children were solicited to collect all information about foods and drinks consumed for separated 3 days [22]. Participants were challenged to memorize and list all the foods and drinks they had consumed the day before, using visuals aids to approximate the serving sizes of various foods:

 
*A Photo Manual (SU.VI.MAX) for Estimating Consumed Portions of Food and Drink* [23].
 
*A Food book: tools to estimate food consumption*; *Aliments et préparations typiques de la population Marocaine* [24].

The quantities consumed were converted to “mg” using the food quantification table, available in the corresponding book, and then entered on the Nutrilog food information software (SAS, version: 2.31.) which was used to translate each food in nutrients and vitamins. In parallel, all details of any vitamin and mineral supplements consumed in the survey period were noted and the mean of the 3 days calcium intake was reported in mg/day. Finally, the average of contribution of dietary sources for total calcium intake was calculated, and values are expressed in g/day in order to estimate exact quantities and in percentages to make comparison with results of the food frequency questionnaire.

### 2.4. Food Frequency Questionnaire

A food frequency questionnaire was also administrated to all participants. Children were asked to complete this questionnaire with complicity of their respective parents and the assistance of a trained investigator. Overall, eight questionnaire items were adjusted to represent foods and beverages frequently consumed by children and adolescents, based upon a review of the data from the study of food intakes for the Moroccan population [25]. The food frequency questionnaire was designed to assess frequency of consumption, over the previous one month, of foods that are reported as good sources of calcium. Utilisation of validated questionnaire is an indispensable tool for estimating dietary calcium intake based on the calcium content in different foods and the frequency of consumption [26].

### 2.5. Statistical Analysis

All analyses were undertaken using the statistical software package IBM SPSS Statistics version 21.0 and Microsoft Office Excel version 2007. Basic descriptive statistics were used to describe each variable. Means were measured for each variable and also to assess many relationships of weight, height, and BMI. Comparisons of variables between males and females were conducted using test chi-square of Pearson. ANOVA test was used to evaluate percentages for independent samples. The normality of the distribution was tested by the Kolmogorov–Smirnov test.

Results are expressed as means ± SD (standard deviation) unless otherwise indicated and *p* value < 0.05 is considered statistically significant. For dietary intake of calcium, children and adolescents were categorized as being at risk of inadequate intake based on whether or not they met the corresponding dietary reference intake recommended by the Institute of Medicine (IOM) [12].

## 3. Results

The study was conducted on 131 participants, 68 boys and 63 girls with a sex ratio of 1.1; the mean age of participants was 10.14 ± 2.5 years. Anthropometric data are reported in Table 1. Overall, participants showed normal anthropometric values with a mean of BMI-*z*-score for age-0.13 ± 1.63. Statistical analysis showed that there was not any significant difference between boys and girls (*p* > 0.05).

The mean calcium intake, calculated according to the 24 h recall protocol, was 521.51 ± 298.06 mg/day, with no significant difference between boys and girls (*p*=0.972). According to the IOM recommendations, children were classified into three age groups: 6 to 8 years, 9 to 13 years, and adolescents between 14 and 18 years [12]. The average of calcium intake according to the age group is reported in Table 2; and clearly shows a high consumption of calcium in subjects aged from 14 to 18 years (776.86 ± 290.07 mg/day) compared to children less than 14 years. Difference of Calcium intake is statistically significant between the different age groups (*p*=0.002). Distribution of calcium intake according to nutritional status, evaluated by BMI-*z*-score (BAZ), is also reported in Table 2. Overall, 70.8% of children have normal nutritional status, and no significant difference is observed (*p*=0.178).

Table 3 provides information of children with inadequate calcium intake. Overall, 85.5% of subjects consume less than recommended adequate intake. Results were reported in both boys and girls, with no distinction of the nutritional status (*p* > 0.05); of particular interest, 100% of obese children have inadequate calcium intake.

**Figure 1:**
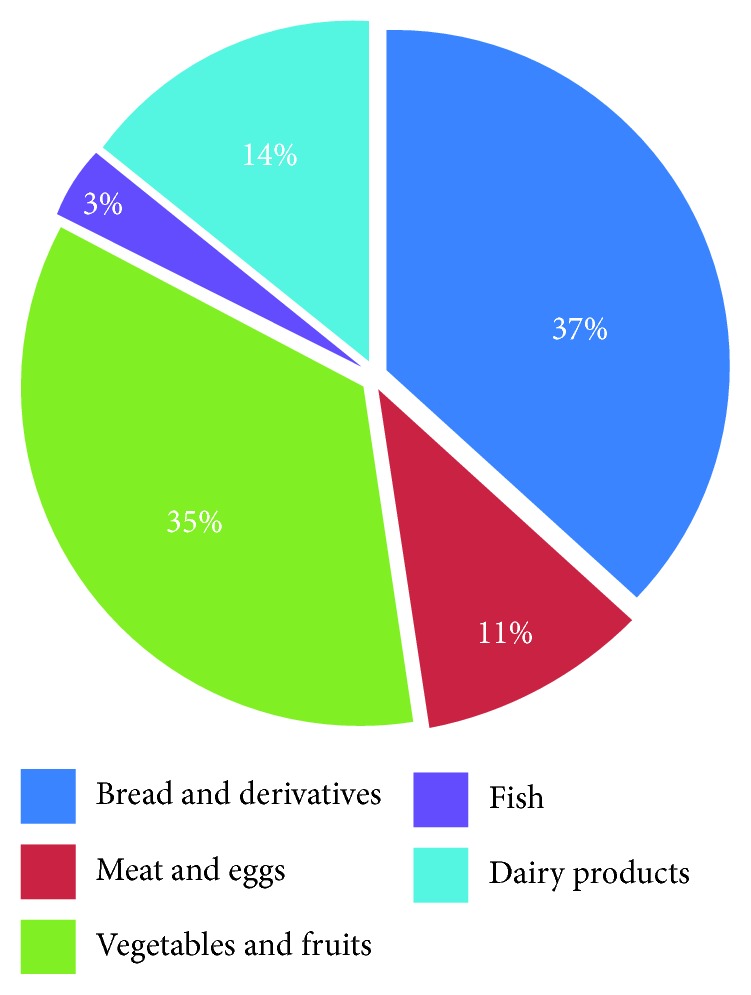
Contribution of dietary sources on total calcium intake (in percentage).

Results from general nutritional and dietary intake are reported in Table 4 and Figure 1. For all findings, statistical analysis showed no significant difference between boys and girls. Concerning daily food consumption, bread, and vegetables and fruits represent the main sources of calcium and contribute to daily intake of 37% and 34%, respectively. Dairy product and meat and eggs are moderately consumed and contribute, respectively, with only 14% and 11% of total daily calcium intake, whereas daily consumption of fish is very low and contribute with only 3% of total calcium intake.

**Figure 2:**
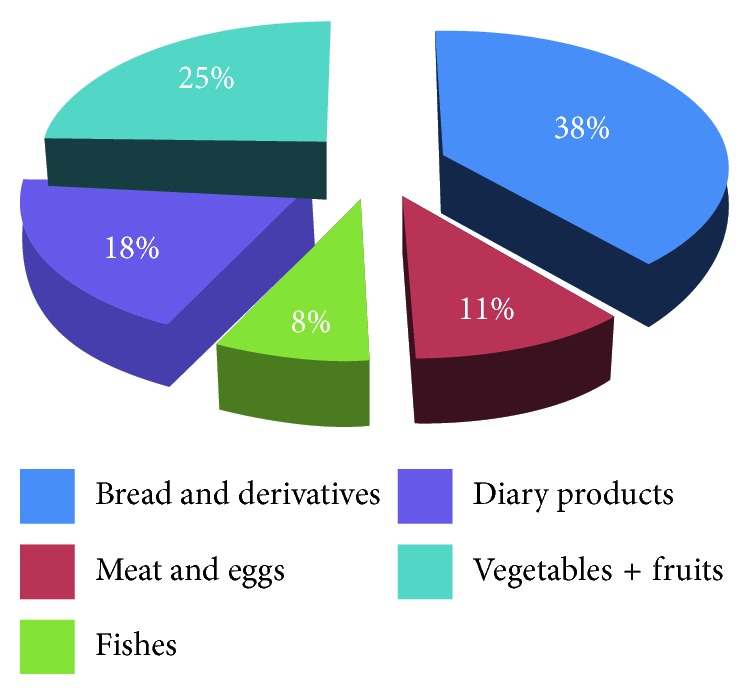
Food frequency distribution.

Food frequency analysis is reported in Figure 2. The choice of food items is limited to foods that are good sources of dietary calcium, and they are expressed in percentage. Results clearly showed that bread and cereals are the main consumed products, representing 38% of total consumed foods. Vegetables and fruits represent 25% whereas meat and eggs represent only 11% of total consumed products. Interestingly, dairy products represent only 18% of daily consumed foods, whereas consumption of fish does not exceed 8%.

## 4. Discussion

The present study was planned to evaluate calcium status in a sample of schoolchildren and adolescents of the Moroccan population as a part of national efforts to prevent health problems related to calcium deficiency. Indeed, and to our best knowledge, there's limited data on calcium status in the Moroccan population, especially on young people where the deficiency is challenging, and its impact is more problematic.

Calcium status is usually assessed directly by dosage on blood samples or indirectly using the 24 h recalls, which is not expensive and the most often used dietary assessment tool in numerous studies [27]. This method has as an advantage rising on its possibility to be used easily with children and adolescents [28]. In this study, 24 h recall (×3) protocol was used to evaluate calcium intake in a population of children and adolescents living in Rabat and neighbour region and showed that total mean calcium intake was 521.51 ± 298,06 mg/day, very lower than the recommended value established by IOM [12]. Moreover, 85.5% of subjects have calcium deficiency. These results are in agreement with those obtained with food frequency analysis highlighting that the usual consumption of products that are good sources on calcium, especially the dairy products, is low.

In Morocco, diet is basically Mediterranean with a large consumption of cereals products, vegetables, and fruits [14]. Moreover, social behaviours and annual incomes are marked by strong disparities between Moroccans, influencing food consumption and diet habits, leading to the low consumption of some foods, including dairy products, meat, and fishes. On the other hand, Morocco, as other developing countries, is facing a nutritional transition marked by the coexistence of nutritional deficiencies and overweight leading to the increase of noncommunicable diseases [29].

These results are in agreement with a previous study conducted in Marrakech, a city in southern Morocco, evaluating calcium intake in subjects aged less than 15 years. In fact, in this study, calcium intake was 839 mg/day, less than recommended values [30]. In this study, food frequency analysis showed that daily consumption of dairy products was considerably higher reaching almost 30% and was the most consumed category of food. These results highlight the difference of eating habits between the two populations and explain the small difference obtained regarding calcium intake.

The calcium status was evaluated in many studies, and the average calcium consumption in children aged between 8 and 10 years was reported to be between 589 and 986 mg/day, whereas for adolescents aged between 10 and 18, the calcium consumption was between 675 and 1273 mg/day [31]. In developing countries, the calcium deficiency is higher: 79% of children aged from 10 to 16 years in Bangladesh and 100% in Senegal [32]. In developed countries, similar studies have reported higher calcium intakes. In USA, Harnack et al. showed that calcium intake in children aged from 11 to 14 years was 993 mg/day [33], and the mean calcium intake evaluated on children aged from 11 to 18 was estimated to be 1172 mg/day [34]. Of particular interest, a multiethnic study on children and adolescents between 10 and 18 years showed that the overall calcium intake was 938 mg/day, but still lower in Asian children with an estimated calcium intake of 868 mg/day, highlighting the cultural behaviours and economic status of their families [35]. In Spain, a study conducted on 8 cities showed that means calcium intake was 859.9 ± 249.2 mg/day [36]. The ANIVA study conducted on children aged between 6 and 9 years showed that 25.8% have calcium deficiency with inadequate calcium intake of 649.44 ±118.11 mg/day whereas normal calcium intake was 1081.10 ±232.79 mg/day [37]. Similar prevalence was also observed in a study done on adolescent girls aged between 12 and 17 years with an average calcium intake limited to 644.4 ± 275 [38].

In this study, no significant difference was found between boys and girls (*p*=0.972). It is widely accepted that calcium concentration is regulated by some hormones, including parathyroid hormones that are different between male and female. However, hormone status is quite similar between boys and girls at childhood. Difference in hormonal status emerges at puberty and is more pronounced at adult age [39, 40]. In addition, we have found a significant association between calcium status and age groups (*p*=0.02), adolescents between 14 and 18 years exhibiting high calcium intake. High calcium status reported in adolescents between 14 and 18 could be explained by the exactitude of given information during interviews compared to those given by children and could be also due to hormonal changes associated with the pubertal period promoting greater mineral utilisation, which needs to be satisfied with suitable calcium consumption [41]. Worldwide, there is limited information on calcium requirements in children and available data converge that calcium retention is low in toddlers and slowly increases as puberty approaches [42]. Moreover, nearly 40% of peak bone mass is acquired during puberty; approximately one-half of total body calcium is laid down during puberty in females and one-half to two-thirds in males [43].

Low calcium intake is a significant health concern and associated with many diseases, increasing the risk of morbidity and mortality [44]. Regulation of calcium intake is widely recommended by different authorities, especially in childhood and adolescents, representing principal periods of growth. Indeed, calcium absorption is optimal at younger age and decreases with age, and usually absorption of calcium is 0.2% less after 40 years [45].

This study gives evidence that calcium status is low and the national program to prevent health problems related to micronutrient deficiencies has to focus its interest on strategies aiming to improve calcium intake through global sensitisation, nutritional education, and promotion of dairy foods. However, the main limitations of the study are the evaluation of calcium intake based only on 24 h recall, with limited sensitivity and specificity and the size of the subsamples obtained after stratification of the total effective of this survey. Therefore evaluation of calcium status using urine and/or blood samples will be of great interest to have a good assessment of calcium intake and to draw strong conclusions and recommendation.

## 5. Conclusion

This study clearly showed that calcium status is very lower in Moroccan children and adolescents and a large proportion of this population have inadequate calcium intake, which depends mainly on an inadequate consumption of calcium-rich products, especially dairy ones. Particular attention must be paid to 9–18 year olds whose increased needs are not being met by current consumption patterns, especially daily products. Hence, specific strategies including children sensitisation and nutritional education are needed to increase calcium intake and therefore reduce calcium deficiency disorders impacting the whole body during childhood and in adult age.

## Figures and Tables

**Table 1:** Anthropometric results of the participants by sex.

	Girls (*n*=63)	Boys (*n*=68)	Total (*n*=131)	*p* value
Age (years)	10.49 ± 2.63	9.82 ± 2.41	10.1 ± 2.54	0.1
Weight (Kg)	34.10 ± 12.43	32.73 ± 11.33	33.39 ± 11.84	0.4
Weight *z*-score	0.0062 ± 1.35197	0.0418 ± 1.71340	0.02 ± 1.55	0.3
Height (cm)	138.56 ± 13.84	137.01 ± 14.42	137.76 ± 14.11	0.53
Height *z*-score	−0.21 ± 1.26	−0.01 ± 1.33	−0.11 ± 1.23	0.75
BMI	17.3 ± 3.47	17.05 ± 3.79	17.17 ± 3.63	0.7
BMI-for-age *z*-score	−0.15 ± 1.38	−0.10 ± 1.83	−0.13 ± 1.63	0.8

*p* values were calculated by one-way ANOVA for means. Results are presented as mean ± standard deviation. BMI (body mass index) and BAZ (BMI *z*-score of body mass index for age) were determined according to [10].

**Table 2:** Distribution of calcium intake according to sex, age groups, and nutritional status.

Variables	*N* (%)	Mean of calcium intake ± SD (mg/day)	*p* value
Sex			
Boys	68 (51.9)	521.13 ± 247.28	**0.972**
Girls	63 (48.1)	521.92 ± 347.43	
Age groups			
6 to 8	45 (34.6)	514.67 ± 247.77	**0.002**
9 to 13	71 (54.6)	475.49 ± 307.06	
14 to 18	14 (10.8)	776.86 ± 290.07	
BAZ			
Thinness	11 (8.5)	705.55 ± 618.05	**0.178**
Normal	93 (70.8)	496.85 ± 249.82	
Overweight	18 (13.8)	541.50 ± 258.63	
Obesity	9 (6.9)	508.67 ± 219.81	
Total	131 (100)	521.51 ± 298.06	

*p* values by One-way ANOVA for means.

**Table 3:** Calcium under recommended adequate intake.

Variables	*N*	Children with inadequate calcium intake^∗^ (%)	*p* value
Sex			
Boys	68	56 (82.35)	0.292
Girls	63	56 (88.89)	
Age groups			
6 to 8	45	35 (77.8)	0.181
9 to 13	71	64 (90.1)	
14 to 18	15	13 (86.7)	
BMZ			
Thinness	11	10 (90.90)	10^–3^
Normal	93	78 (83.87)	
Overweight	18	15 (83.33)	
Obesity	9	9 (100)	
Total	131	112 (85.50)	

*p* values are calculated by test chi-square of Pearson. ^∗^Results reported as IOM recommendations for adequate intake [12].

**Table 4:** Dietary intake according to sex.

	Total	Boys	Girls	*p* value
Nutritional intake				
Total energy intake (kcal/day)	1887.19 ± 929.01	1948.9 ± 1018.27	1818.52 ± 821.18	0.425
Protein (g/day)	63.1 ± 29.10	72.82 ± 30.37	63.90 ± 29.10	0.093
Fat (g/day)	64.31 ± 44.87	66.75 ± 53.11	61.61 ± 33.69	0.515
Sugars (g/day)	249.97 ± 116.97	260.61 ± 124.72	238.12 ± 107.46	0.273
Dietary fiber (g/day)	24.37 ± 12.50	25.40 ± 10.59	23.2294 ± 14.34057	0.322
Total water (g/day)	3061.45 ± 658.15	3089.60 ± 677.86	3030.11 ± 639.54	0.607
Total food (g/day)	1749.29 ± 595.33	1827.0 ± 639.63	1662.68 ± 533.65	0.115
Dietary intake (g/day)				
Bread and derivatives	365.05 ± 231.53	390.61 ± 263.53	336.61 ± 187.79	0.184
Meat and eggs	106.06 ± 88.42	114.03 ± 104.58	97.19 ± 65.73	0.278
Vegetables and fruits	345.63 ± 226.08	344.48 ± 166.10	346.9 ± 279.6	0.951
Fish	31.52 ± 49.85	35.62 ± 44.98	26.952 ± 54.78	0.322
Dairy products	140.89 ± 107.44	139.12 ± 111.08	142.87 ± 104.11	0.843

*p* values by one-way ANOVA for means. Results are presented as mean ± standard deviation.
